# MXenes *à la Carte*: Tailoring the Epitaxial Growth Alternating Nitrogen and Transition Metal Layers

**DOI:** 10.1021/acsnano.2c04029

**Published:** 2022-07-22

**Authors:** José D. Gouveia, Ángel Morales-García, Francesc Viñes, José R. B. Gomes, Francesc Illas

**Affiliations:** † Departament de Ciència de Materials i Química Física & Institut de Química Teòrica i Computacional (IQTCUB), Universitat de Barcelona, c/Martí i Franquès 1-11, 08028 Barcelona, Spain; ‡ CICECO − Aveiro Institute of Materials, Department of Chemistry, University of Aveiro, Campus Universitário de Santiago, 3810-193 Aveiro, Portugal

**Keywords:** MXenes, epitaxial growth, density functional simulations, thermodynamics, kinetics

## Abstract

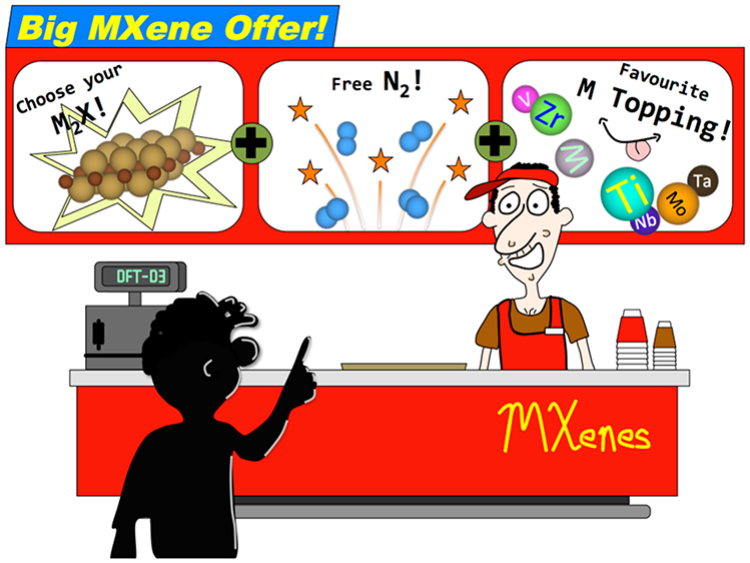

A high-throughput analysis based on density functional simulations underscores the viable epitaxial growth of MXenes by alternating nitrogen and metal adlayers. This is supported by an exhaustive analysis of a number of thermodynamic and kinetic thresholds belonging to different critical key steps in the course of the epitaxial growth. The results on 18 pristine N- and C-based MXenes with M_2_X stoichiometry reveal an easy initial N_2_ fixation and dissociation, where N_2_ adsorption is controlled by the MXene surface charge and metal d-band center and its dissociation controlled by the reaction energy change. Furthermore, formation energies indicate the plausible formation of N-terminated M_2_XN_2_ MXenes. Moreover, the further covering with metal adlayers is found to be thermodynamically driven and stable, especially when using early transition metal atoms. The most restrictive analyzed criterion is the N_2_ adsorption and dissociation at nearly full N-covered adlayers, which is yet achievable for almost half of the explored M_2_X seeds. The present results unfold the possibility of expanding, controlling, and tuning the composition, width, and structure of the MXene family.

MXenes are two-dimensional (2D) materials^1^ which have recently attracted a considerable amount of attention because of their possible technological uses in a large number of fields,^2^ ranging from adequate substrates for future generations of lithium-based batteries,^3^ to effective materials for electromagnetic interference (EMI) shielding,^4^ and as suitable substrates for carbon dioxide (CO_2_) capture,^5−7^ a key issue in the quest to fight global warming. Furthermore, many research endeavors have put MXenes under the spotlight as sensors for organic or biological compounds,^8−10^ up to the point that MXene–graphene composites have been indicated as low-cost, ultrasensitive, and fast-responding sensors for the H1N1 influenza virus and SARS-CoV-2 coronavirus.^11^

All of the above MXene uses highly depend on their composition, geometry, and surface termination. Most MXenes can be classified as 2D few-layered transition metal carbides and nitrides with a M_*n*+1_X_*n*_ formula, with *n* defining the thickness of the MXene, with normally *n* = 1–3, although *n* = 4 MXenes, such as Mo_4_VC_4_,^12^ have been synthesized and characterized, and there are reports of trace impurities of MAX phases with larger *n*, such as Ta_6_AlC_5_ and Ti_7_SnC_6_.^13,14^ The M component is generally an early transition metal, typically from groups III–VI of the Periodic Table, while X is either C or N. This already generates a large pool of combinatorial possibilities, but the family size is further increased when accounting for carbonitride MXenes,^15^ as well as bimetallic MXenes, either in the form of layered solid solutions (*i*-MXenes) or displaying some degree of layered ordering (*o*-MXenes).^16^

Additionally, one has to account for the MXene surface terminations, generally denoted as T_*x*_, resulting from the MXene synthesis protocol, which considerably enlarge the aforementioned pool of possibilities. MXenes are generally attainable by selective chemical etching of the A element of MAX phases, usually employing hydrofluoric acid (HF) for such a purpose,^1^ followed by sonication to separate the MXene layers, although the use of lithium fluoride (LiF) and hydrochloric acid (HCl) to generate *in situ* HF has been gaining momentum.^17^ This treatment generally leaves the MXene surface terminated by a combination of hydrogen (H), oxygen (O), hydroxyl (OH), and fluorine (F) groups for a variety of working conditions,^18,19^ despite diverse fluorine-free synthesis procedures have been proposed to control the variable surface termination and ease the preparation process.^20,21^

Last but not least, there have been recent advances showing that pristine MXenes, *i.e.*, with no surface terminations, can be gained, either from a T_*x*_-containing one, removing the T_*x*_ after hydrogenation and annealing protocols,^7^ or directly synthesized departing from halide-terminated MXenes.^22^ All in all, the MXene family is vast and quickly growing. However, their number is quite restricted to the availability of a suited MAX phase —over 155 so far reported and growing, including some *n* > 3 materials.^23^ Within this context, apart from grinding synthetic methods, one has to highlight the possibility of gaining MAX thin films epitaxially grown on a given support, such as sapphire Al_2_O_3_, by magnetron sputtering,^24,25^ with reports revealing the synthesis of bimetallic alloy MAX phases such as (Ti,Zr)AlC_2_^26^ or the Ge-based Ti_*n*+1_GeC_*n*_ (*n* = 1–3) family.^27^

Spurred by these previous findings, a (still open) question arises: Would it be feasible to directly epitaxially grow MXenes, even to go for *n* > 3? To answer this question, one must obviously regard the recently experimentally gained pristine MXenes,^7,22^ as the surface T_*x*_ would act as a passivator layer. Within this frame, it has been shown, by means of density functional theory (DFT) computational studies, that pristine M_2_X MXenes —M = Ti, Zr, Hf, V, Nb, Ta, Cr, Mo, W; X = C or N— are quite capable of chemically adsorbing nitrogen (N_2_), with adsorption energies, *E*_ads_, ranging from −1.11 to −3.45 eV, and dissociating it with N_2_ dissociation energy barriers, *E*_b_, between 0.28 and 1.10 eV.^28^ There exist metal chemical vapor deposition (CVD) methods to create early M films, mostly using a transition metal halide or complex as a precursor.^29^ Actually, MBr_4_ and MCl_4_ compounds have been used to generate Cl- or Br-terminated MXenes, and given the weak M–halide bond, these have been used to obtain pristine MXenes as well as a large variety of additional T_*x*_ terminations (T_*x*_ = O, S, NH, Se, Te).^22^

With the information provided above, one could envisage a process in which a pristine MXene M_2_X seed material is used to capture and decompose N_2_, ideally eventually gaining a fully N-covered M_2_XN_2_ MXene surface, and that this could, in turn, be used as well as a substrate to deposit M′ layers on top, resulting in M′_2_M_2_XN_2_ compounds, where M′ could be, in principle, any early transition metal compound. Lastly, by combining these processes, one would effectively epitaxially grow the MXene compound while controlling the composition of the outer layers. Hereby, we demonstrate, by DFT-based modeling on a series of MXene systems, that such epitaxial growth is attainable for several MXene seeds, meeting a number of thermodynamic and kinetic thresholds and backing up the tailor-making of MXenes with a controlled surface metal M and X ending.

## Decalogue of Stability

In order to rationalize the suitability of epitaxially growing MXenes in terms of thermodynamic and kinetic stability, a list of 10 criteria was defined, as disclosed below, following natural steps to be successively met in the course of the epitaxial growth; for instance, N_2_ has to chemically adsorb on the studied M_2_X MXene seed prior to its molecular dissociation. Thus, epitaxial growth is likely to happen when the 10 criteria are met in succession, although all criteria have been examined for all of the cases. This was not done just for completeness but also as one could envisage alternative paths to ensure accomplishing one unmet criterion, which acts as a pebble-in-the-shoe in the epitaxial growth; for instance, we consider N_2_ as the N source, but it could well be ammonia, pyridine, or pyrrole, to name a few. The goal of this evaluation is thus to provide a roadmap on the epitaxial growth, although secondary paths are not excluded from the equation.

The 10 criteria are as follow, so, for a given M_2_X to be a likely good seed for epitaxial growth, it must display:

(*i*) A thermodynamically favorable N_2_ adsorption on pristine M_2_X. For this, the N_2_ adsorption energy, *E*_ads_^N_2_^, is defined as
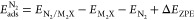
1where *E*_N_2_/M_2_X_ is the total energy of the M_2_X MXene with a N_2_ molecule adsorbed on one of its (0001) surfaces, *E*_M_2_X_ is the total energy of the corresponding M_2_X pristine MXene, and *E*_N_2__ is the energy of the isolated N_2_ molecule. The Δ*E*_ZPE_ term is the difference in zero-point energy (ZPE) of the N_2_ in the gas phase or when adsorbed. According to this definition, a favorable adsorption implies negative *E*_ads_^N_2_^ values, and hence, the more negative, the stronger the adsorption is. Note that for N_2_ in a vacuum, there is only one vibrational frequency related to the N_2_ bond stretching. However, when adsorbed, the linear symmetry is broken and the rotational and translation normal modes become frustrated due to the bonding of the N_2_ molecule to the MXene substrate. Therefore, the number of normal vibrational modes of the adsorbed species becomes six. That considered, the ZPE for gas-phase species or when adsorbed are calculated as
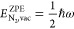
2
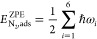
3respectively, where ℏ is the reduced Planck constant and ω_*i*_ are the vibrational angular frequencies. Thus, the ZPE term is simply
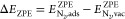
4

(*ii*) A kinetic preference toward N_2_ dissociation compared to the N_2_ desorption. In this regard, the N_2_ dissociation energy barrier on the pristine M_2_X MXene seed (0001) surface, *E*_b_^N_2_^, should be by default smaller than the N_2_ molecular desorption energy, *E*_des_^N_2_^, here defined as the inverse of the adsorption energy, i.e., *E*_des_^N_2_^ = −*E*_ads_^N_2_^, succinctly implying that dissociation is easier than desorption.

(*iii*) An energetic preference towards N_2_ dissociation. The N_2_ dissociation reaction step energy difference, Δ*E*_dis_^N_2_^, calculated as the energy difference between the final dissociated state containing two vicinal N adatoms and the initial state having the adsorbed N_2_ molecule has to be clearly exothermic, implying that there is a tendency toward its dissociation rather than toward the recombination of the two N adatoms into adsorbed N_2_.

So far, criteria (*i*–*iii*) would define the likeliness of pristine M_2_X MXenes in capturing and breaking a N_2_ molecule, eventually having a MXene with two vicinal N adatoms, i.e., having MXenes with an N-adatom surface coverage, θ_N_, of ^2^/_9_ monolayers (ML), where the number 2 is due to the dissociated N_2_ molecule, and 9 is the number of active sites in our *p*(3×3) MXene supercell model, equal to the number of atoms on each of the MXene exposed surfaces. The next criteria evaluate these and other aspects for the same process but to eventually obtain an M_2_X MXene with full N-adatom coverage, i.e., θ_N_ = 1 ML. To this end, a full N-coverage situation was designed for each studied M_2_X, acquiring and optimizing the atomic structure of M_2_XN_2_ models, departing, as aforementioned, from most stable stacking situations. The stability of the resulting M_2_XN_2_ MXenes was further analyzed, beyond ABC and ABA stacking, in order to find the most stable minimum, implying, in some cases, different N-terminations, *e.g.*, an NMXMN stacking following a BABCA sequence, as found on Ti_2_CN_2_, where both surface N layers have different environment, *i.e.*, being aligned with the central X layer or with the farthest M layer. Such cases are Janus MXenes by definition,^30,31^ where both surface endings are different. In such cases, the following criteria were evaluated on each surface ending. Thus, once the structure is optimized, two vicinal N adatoms are removed and the structure reoptimized, so that a model with θ_N_ = ^7^/_9_ ML is created. For a favorable epitaxial growth, these models must display:

(*iv*) a thermodynamically favorable N_2_ adsorption,

(*v*) a kinetic preference toward N_2_ dissociation compared to N_2_ desorption,

(*vi*) an energetic preference toward N_2_ dissociation.

These are the same as criteria (*i*–*iii*), but for a nearly full N-coverage, altogether fringing the limits of a full monolayer creation, *i.e.*, kinetically and energetically suitable from its first stage up to the final full-coverage situation. Further than that, the as-created M_2_XN_2_ MXene must have the following:

(*vii*) a suitable formation energy for M_2_XN_2_. To this end, the formation energy, *E*_form_, is defined as
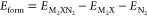
5where *E*_M_2_XN_2__ is the energy of the optimal M_2_XN_2_ MXene, and the *E*_form_ values obtained are given per formula unit. In this context, a suitable formation energy must be negative, implying that the formation of M_2_XN_2_ is exothermic and thermodynamically driven.

Finally, on the so-gained M_2_XN_2_, one may adsorb a single early transition metal M′ atom from groups IV–VI, with θ_M′_ = ^1^/_9_ ML, and also envision a full-coverage situation, θ_M′_ = ^9^/_9_ ML, in which such atoms occupy most stable positions on the surface —or on each surface in case of Janus M_2_XN_2_ models. In the latter case, the atomic structure of the resulting M′_2_M_2_XN_2_ MXene is again fully optimized, and on such a model, an M′ atom is removed and the system structure reoptimized to have a nearly full M′ surface situation, θ_M′_ = ^8^/_9_ ML. On these models, one has to evaluate whether M_2_XN_2_ is suited for an epitaxial growth, as they must display the following:

(*viii*) A viable metal adatom adsorption energy on pristine M_2_XN_2_. In this sense, the metal adatom adsorption energy, *E*_ads_^M′^, is calculated as
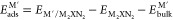
6where *E*_M′/M_2_XN_2__ is the energy of the M_2_XN_2_ model with one M′ adatom adsorbed, and *E*_bulk_^M′^ is the bulk energy of a single M′ atom. Notice that bulk energies had already been obtained in previous DFT studies using the same computational setup.^59−61^ Within this definition, a negative *E*_ads_^M′^ value implies that the single atom is energetically more comfortable as an adatom on the M_2_XN_2_ model than in its pure M′ bulk environment, a thermodynamic assessment that has long been used in seizing the single-atom surface thermodynamic stability on 2D materials.^32−34^ Note that this criterion is more stringent than when the energy of the M′ atom is assumed to be the energy of the isolated M′ atom in vacuum.

(*ix*) A viable metal adatom adsorption energy to form M_2_′M_2_XN_2_. This is, a negative *E*_ads_^M′^ value when adsorbed on the θ_M′_ = ^8^/_9_ ML M_2_′M_2_XN_2_ model; in other words, on the M′ surface vacancy of the otherwise fully M′ covered M_2_′M_2_XN_2_ model. This threshold implies that such a full metal monolayer is thermodynamically driven. This adsorption energy is obtained as
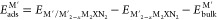
7where *E*_M′_2–*x*_M_2_XN_2__ is the energy of a θ_M′_ = ^8^/_9_ ML M′_2_M_2_XN_2_ model, and *E*_M^′^/M′_2–*x*_M_2_XN_2__ the energy of a θ_M′_ = 1 ML M_2_′M_2_XN_2_ model.

(*x*) A suitable formation energy for M′_2_M_2_XN_2_. To this end, the M′_2_M_2_XN_2_ formation energy is calculated as
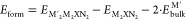
8where *E*_M^′^/M′_2_M_2_XN_2__ is the energy of a θ_M′_ = 1 ML M′_2_M_2_XN_2_ model, and given per M′_2_M_2_XN_2_ formula unit. Thus, a negative *E*_form_ would imply that the formation of M′_2_M_2_XN_2_ from bulk M′ and M_2_XN_2_ would be exothermic, and thermodynamically favorable. Thus, criteria (*viii*–*x*) define whether the formation of M′ adlayers is thermodynamically driven.

## Results and Discussion

**Figure 1:**
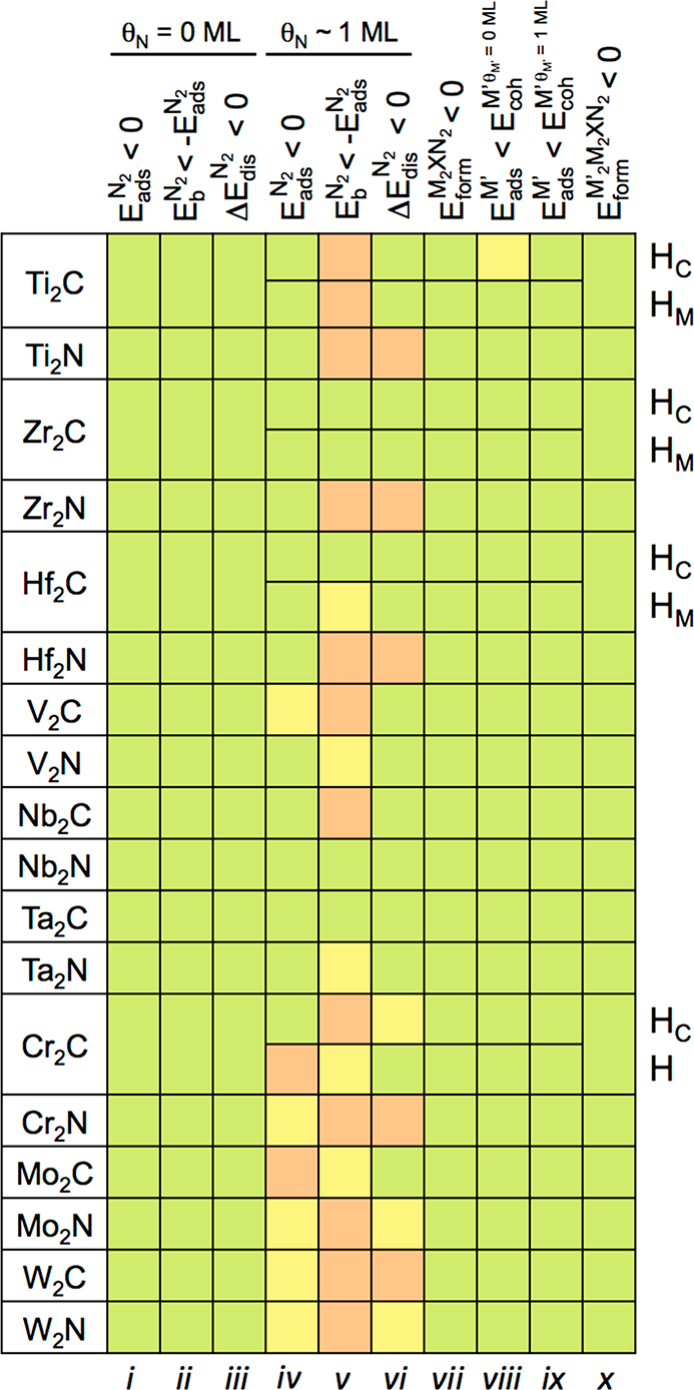
Summary of accomplishment of the 10 (*i*–*x*) decalogue criteria for epitaxial growth alternating N and M′ adlayers over a M_2_X central MXene seed. The upper tags represent the followed criteria at certain given coverage conditions. Right tags denote different surface endings for some M_2_XN_2_ models. Light green (red) color implies meeting (or not) the sought criterion, while light yellow color implies dubious cases within the DFT ± 0.2 eV accuracy range. H_C_ and H_M_ denote hollow sites where the adatoms are vertically aligned with a carbon or metal, respectively.

Having defined the decalogue of conditions enabling an epitaxial growth of alternating N and M′ layers on an M_2_X MXene seed, we analyze them one at a time in this section. Let us begin with the N_2_ molecule adsorption energy on the pristine M_2_X (0001) surface models. The computed adsorption energies for all the models have been collected from the literature,^28,53^ taking into account the most preferred M_2_X layers stacking. For convenience, the *E*_ads_^N_2_^ values are listed in Table S1 of the Supporting Information (SI). The casuistry of this criterion is graphically summarized in Figure 1, assuming that any negative *E*_ads_^N_2_^ value is favorable and, *vice versa*, that any positive *E*_ads_^N_2_^ value is unfavorable. Given the standard DFT accuracy of ±0.2 eV, *i.e.*, assuming that *E*_ads_^N_2_^ values may vary by this amount when using another exchange-correlation functional and/or estimate of dispersive forces correction, *E*_ads_^N_2_^ values of ±0.2 eV are taken with a grain of salt, and considered dubious. In the present situation, notice that, since *E*_ads_^N_2_^ values range from −1.14 (W_2_C) to −3.45 eV (Ti_2_N), in all cases indicating a strong chemisorption. Thus all of the M_2_X seed accomplish this first criterion. As can be seen in Figure S1 of the SI, the *E*_ads_^N_2_^ values on pristine M_2_X models seem to correlate with both the surface metal *d*-band center, ε_d_, and the surface metal charge, Δ*Q*_m_, with regression coefficients, *R*, of 0.76 and 0.83, respectively, and so, governed by the same factor that determines CO_2_ and CO adsorption strengths, as previously reported.^35^

The second criterion is that the dissociation energy barrier for the adsorbed N_2_, *E*_b_^N_2_^, must be smaller than the desorption energy, here defined as −*E*_ads_^N_2_^. Table S1 of the SI collects the computed *E*_b_^N_2_^ values from the literature,^28,53^ ranging from 0.18 (W_2_N) to 1.1 eV (Zr_2_C). Even considering the most detrimental error thresholds due to the DFT accuracy, that is, *E*_ads_^N_2_^ values systematically overestimated, and *E*_b_^*N*_2_^ values systematically underestimated, the difference between the estimated *E*_ads_^N_2_^ and *E*_b_^N_2_^ values is large, at least of 0.74 eV for Mo_2_C, implying that this criterion is accomplished for all the investigated M_2_X MXenes; see Figure 1. Finally, the third criterion for the pristine M_2_X seeds is that the N_2_ dissociation reaction step energy difference, Δ*E*_dis_^N_2_^, should be negative (exothermic). The values, taken again from the literature,^28,53^ and encompassed in Table S1 of the SI, are all negative, ranging from −1.55 (Ti_2_C) to −3.22 eV (W_2_N). Thus, even when accounting for DFT accuracy, all of the explored MXenes accomplish this last criterion, as seen in Figure 1.

**Figure 2:**
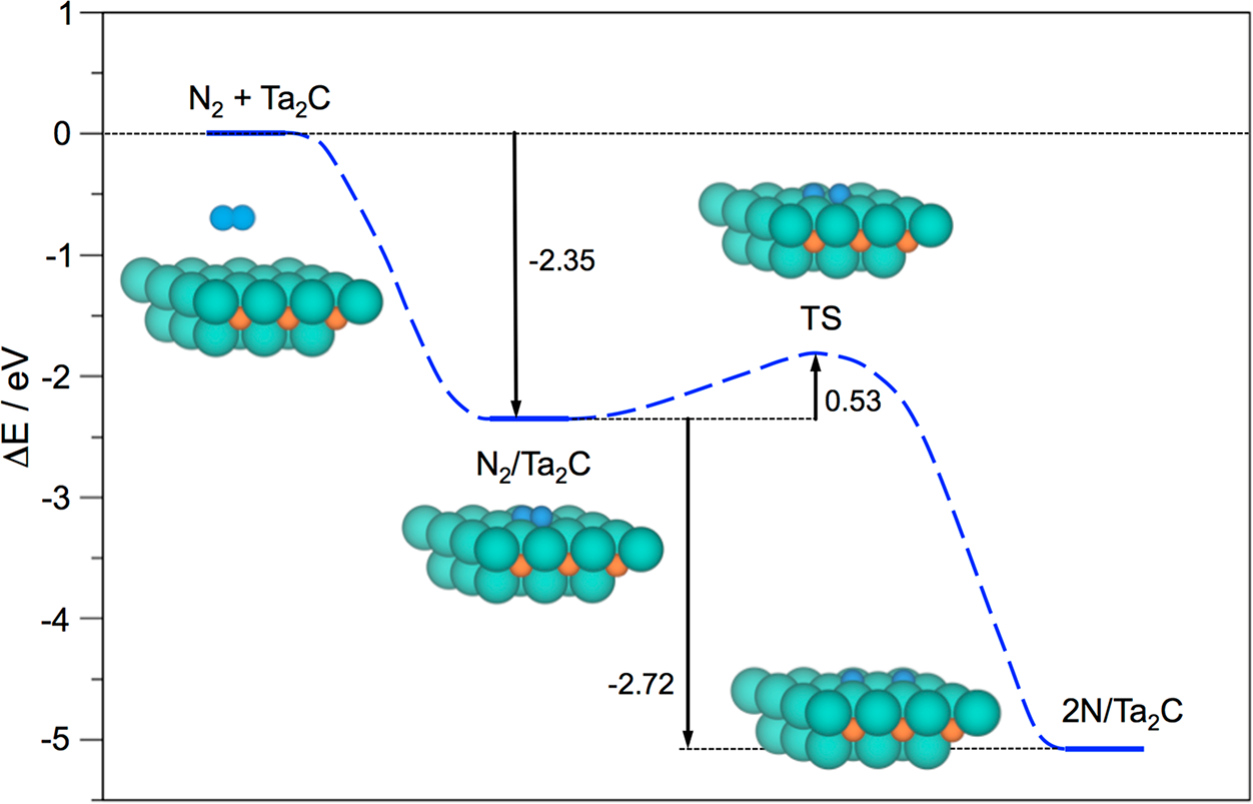
Reaction energy profile for N_2_ adsorption on the pristine Ta_2_C (0001) surface model and its subsequent dissociation into two N adatoms, overcoming the reaction step transition state. The atomic models display the different stages of the process, with Ta, C, and N atoms represented by light green, light brown, and blue spheres, respectively.

As a representative example, Figure 2 depicts the reaction profile for the N_2_ adsorption and dissociation steps on the Ta_2_C (0001) model. Notice here how the *E*_ads_^N_2_^ of −2.35 eV is clearly larger in magnitude than the N_2_ dissociation energy barrier, *E*_b_^N_2_^, of 0.53 eV. Finally, the N_2_ dissociation elementary step implies a favorable energy reduction, Δ*E*_dis_^N_2_^, of −2.72 eV. Thus, all of the first three criteria of the decalogue are met. Notice that the *E*_b_^N_2_^ and Δ*E*_dis_^N_2_^ values follow a clear Brønsted–Evans–Polanyi (BEP) relationship, including to this end high-coverage situations, see below and Figure S2 of the SI, with a regression coefficient, *R*, of 0.96, and a mean absolute error on predicted *E*_b_^N_2_^ of solely 0.3 eV, close to the aforementioned DFT accuracy.

Further than this, we evaluated the working conditions at which N adatom moieties, N*, are kinetically preferred to be located at the Ta_2_C (0001) surface. Accordingly, adsorption/desorption rates, as well as N_2_ dissociation and 2N recombination rates have been estimated as a function of temperature, *T*, and N_2_ partial pressure, *p*_N_2__. Briefly, collision theory was used for the adsorption rate, while transition state theory was used for the rest of the reaction elementary steps, using a N_2_ sticking coefficient similar to that of N_2_ on Pt surfaces, of 0.68,^36^ also justified by the similar behavior of early transition metal carbides to Pt group elements.^37^ Aside, a rotational temperature of 2.88 K was employed for N_2_ in vacuum, as taken from the literature,^38^ while nine adsorption sites are considered on each exposed MXene surface area. For more details on the mathematical framework and procedure, we refer the reader to the literature.^35^

**Figure 3:**
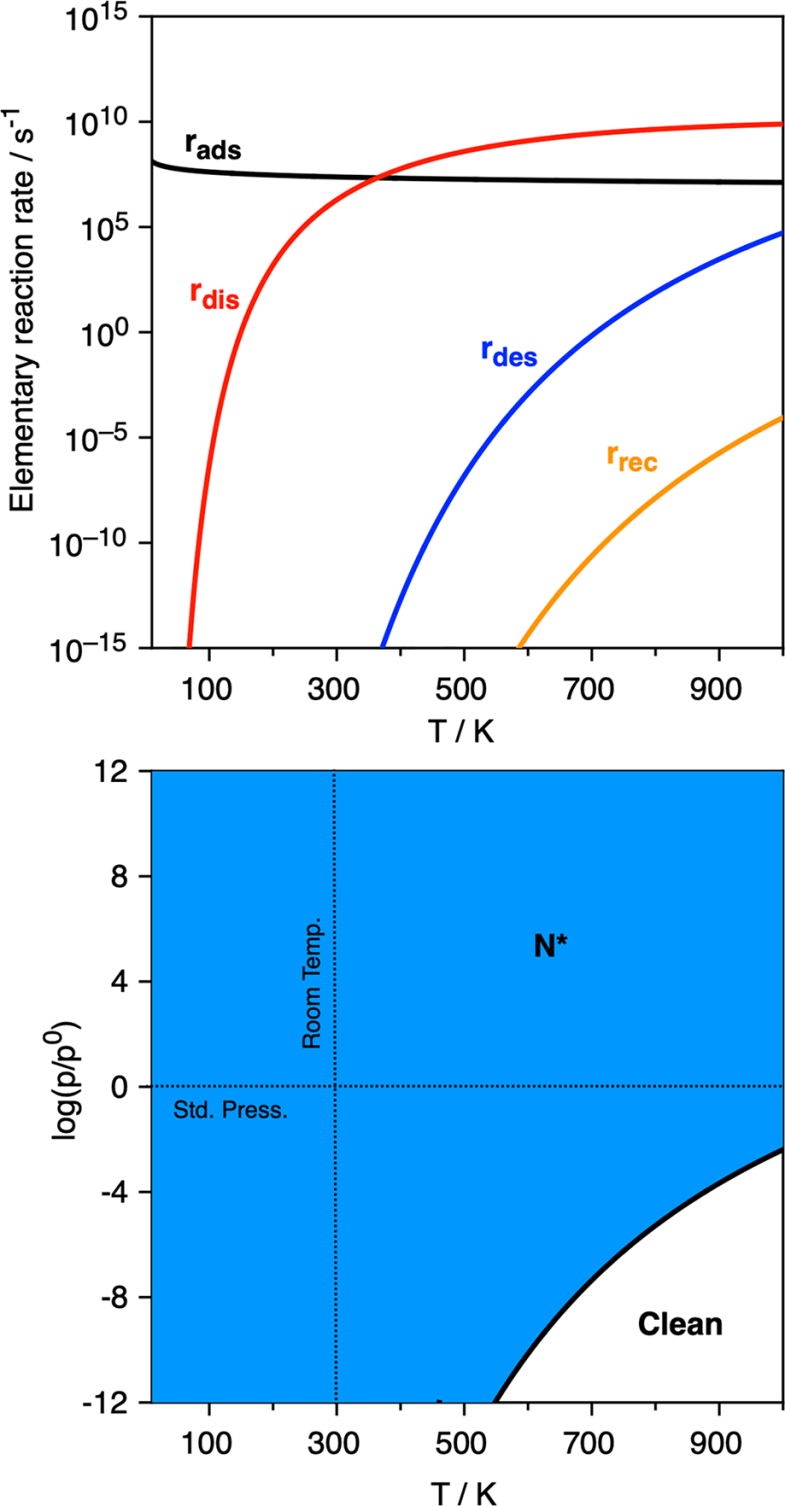
Top panel: Elementary reaction rates on Ta_2_C (0001) surface for the N_2_ adsorption at 0.1 bar as a function of temperature, *T*. The *r*_ads_ and *r*_des_ quantities stand for adsorption and desorption rates, respectively, whereas *r*_dis_ and *r*_rec_ correspond to the N_2_ dissociation and 2N recombination rates, respectively. Bottom panel: Kinetic phase diagram for N_2_ adsorption and dissociation as a function of N_2_ partial pressure, *p*_N_2__, and *T*. Dark blue colored areas reveal regions of preference toward the N* moiety.

Figure 3 shows the different elementary reaction step rates and the built kinetic phase diagram, where a solid line denotes the equilibrium conditions where *r*_ads_ = *r*_des_, so that for *T* and *p*_N_2__ values under the line, the desorption process is preferred over the adsorption one, and the system would have a preference toward being clean, *i.e.*, absent of adsorbed atomic or molecular moieties.^39^ On the other hand, for *p*_N_2__ and *T* conditions above the equilibrium line, the N_2_ adsorption rate, *r*_ads_, is larger than the desorption rate, *r*_des_, implying a preferential accumulation of N_2_ on the Ta_2_C (0001) surface. Moreover, within this region, the molecularly adsorbed N_2_, N_2_*, dissociation rate, *r*_dis_, is always larger than the 2N recombination rate, *r*_rec_, and so N* adatoms are envisioned as the kinetically driven surface species. All in all, N_2_ adsorption and dissociation seems quite feasible on the Ta_2_C (0001) selected model, as well as on other studied MXenes. For instance, on Ta_2_C, and with N_2_ at standard pressure; *p*_N_2__ = 1 bar, one could start accumulating N* moieties at working temperature below the 1000 K explored limit. However, one should regard that such conditions are suited for the initial stages of N covering, but they may change for larger θ_N_, *vide infra*.

**Figure 4:**
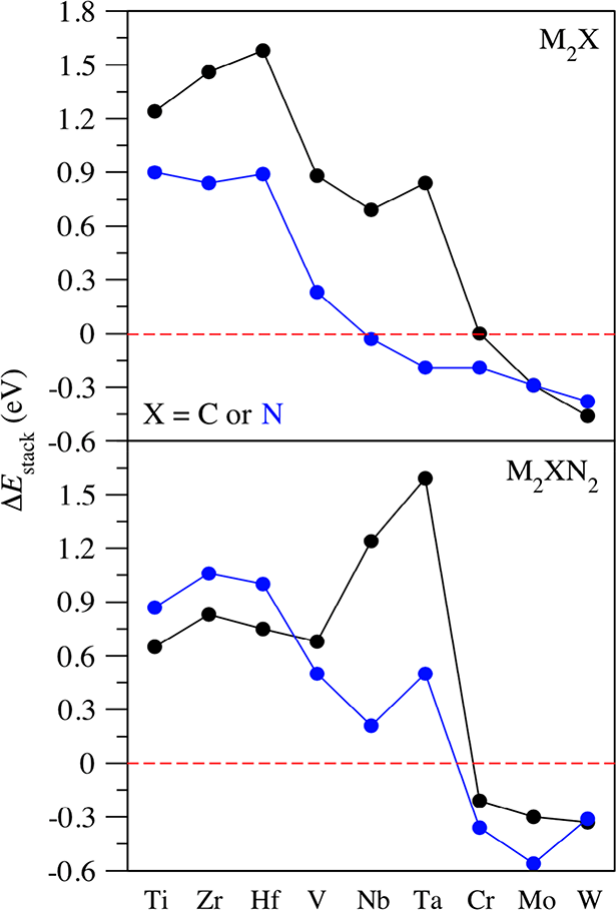
Plots of Δ*E*_stack_ as a function of the M element for X = C (black) or N (blue) M_2_X seed compositions, either for pristine (top panel)^53^ or N-terminated (bottom panel) models. The red dashed line denotes equal energetic stability between ABC and ABA stackings, with negative values indicating preference for the latter.

Accordingly, we next explored the fully N-covered M_2_X, *i.e.*, M_2_XN_2_ models. The structural optimizations considering different possible stacking options revealed that, similarly to the previous observations on O-terminated MXenes,^53^ the group VI (Cr_2_X, Mo_2_X, and W_2_X) MXenes featured a thermodynamic preference toward ABA stacking; see the difference in energy of ABA versus ABC per formula unit, Δ*E*_stack_, in Figure 4 and values in Table S2 of the SI. Notice that, compared to the case of O-termination, the N-termination still favors ABA stacking, but by a smaller energy difference, this is, Δ*E*_stack_ values ranging from −0.21 (W_2_CN_2_) to −0.56 eV (Mo_2_N_3_) are significantly smaller when compared to the values of −1.05 (Cr_2_CO_2_) to −2.33 eV (W_2_CO_2_), as reported in previous work.^53^

Contrary to that previously stated, the N-termination does not necessarily have to follow the stacking trend. For instance, structural optimizations revealed a different surface ending for group IV carbon-based MXenes (Ti_2_C, Zr_2_C, Hf_2_C), where the N surface termination layers occupy different surface sites on each face of the material; in particular, adsorbed on a hollow site aligned with a metal atom lying three layers underneath (H_M_), or adsorbed on a hollow site but aligned with a C atom two layers underneath (H_C_). This means that Ti_2_CN_2_, Zr_2_CN_2_, and Hf_2_CN_2_ models are actually Janus MXenes, with different surface terminations, and consequently, each one was explored separately. A similar situation occurs for Cr_2_CN_2_, but here, given the ABA stacking of the Cr_2_C seed, the N layers occupy either H_C_ or empty hollow (H) sites. It was noticed that such Janus structures are not observed on M_2_N seeds, succinctly suggesting that such different surface terminations come from having alternate C and N layers across the MXene structure.

Having defined and optimized the structure of M_2_XN_2_ models, two neighboring N vacancies were generated on one of the two surfaces —or the different surface endings in the case of Janus models, and the resulting model was used to investigate the N_2_ adsorption energy for θ_N_ = ^7^/_9_ ML, explored on the N-free surface patch. The calculated adsorption energies are listed in Table S3 of the SI and reveal that, in general terms, such surface N-free patches maintain a chemical affinity toward N_2_, with negative *E*_ads_^N_2_^ values going from −0.43 (Zr_2_N) to −2.05 eV (Ta_2_N). However, on this fourth criterion, one starts finding certain cases where a full N-coverage is not likely to be met. For instance, Mo_2_C shows an adverse *E*_ads_^N_2_^ of 0.97 eV. Further than that, group VI N-based MXenes, Cr_2_N, Mo_2_N, and W_2_N, as well as W_2_C and V_2_C, still feature negative N_2_ adsorption energies yet weaker than −0.2 eV and so are catalogued as dubious cases in Figure 1.

Two more surprising trends emerge within this criterion; first, at variance with the θ_N_ = 0 ML results shown in Table S1 of the SI, the N-based group V MXenes (V_2_N, Nb_2_N, Ta_2_N) display sensibly stronger *E*_ads_^N_2_^ values, fringed between −0.87 (V_2_N) and −2.05 eV (Ta_2_N), when compared to those of C-based seeds, going from −0.2 (V_2_C) to −1.20 eV (Ta_2_C). The second curious feature is that Cr_2_C, displaying a Janus structure, shows a noted distinct N_2_ affinity depending on whether the N adatoms occupy H_C_ or H sites, going from a markedly exothermic adsorption of −1.65 eV on the H_C_ ending, to a notably endothermic adsorption of 0.5 eV on the H ending. This distinct chemical activity could imply that a θ_N_ = 1 ML can be reached on the H_C_ side, while only a partial θ_N_ could be achieved on the other H surface ending.

When it comes to the N_2_ dissociation energy barrier at a near θ_N_ = 1 ML situation, one readily observes in Figure 1 that this is indeed the most stringent explored criterion, not met in 10 out of 18 MXenes. The values listed in Table S3 of the SI reveal cases with high *E*_b_^N_2_^ values, such as group IV M_2_N seeds, that is, Ti_2_N, Zr_2_N, and Hf_2_N, with barriers surpassing 5.4 eV. These prohibitive barriers are the result of moderate *E*_ads_^N_2_^ values not exceeding −0.53 eV, basically depicting a physisorption state in which the N_2_ molecular bond is neither compromised nor activated toward its breakage. This is to be put together with large Δ*E*_dis_^N_2_^ values above 4.1 eV (see discussion below), implying that the reaction step is thermodynamically quite endothermic and, concomitantly, requires surpassing large energy barriers. The rest of the estimated *E*_b_^N_2_^ are more moderate, framing very likely situations, such as the barrier of solely 0.24 eV of Nb_2_N to sensibly large values such as the 2.81 eV for the H_C_-terminated surface on Cr_2_C. In any case, the *E*_b_^N_2_^ values have to be compared to the desorption energy values and, in this regard, only Zr_2_C, Nb_2_N, Ta_2_C, and the H_C_ surface of Hf_2_C, yet closely followed by dubious cases of V_2_N, Ta_2_N, Mo_2_C, and H_M_ surface termination on Hf_2_C, and H termination of Cr_2_C can be considered viable.

The exothermic Δ*E*_dis_^N_2_^ criterion is, in comparison, achieved in a larger number of cases. Apart from the just mentioned group IV M_2_N cases, only Cr_2_N (1.32 eV) and W_2_C (0.68 eV) feature clearly endothermic situations, and basically isoenergetic situations are found for H_C_ surface termination of Cr_2_C, plus Mo_2_N and W_2_N. Other than these cases, the N_2_ dissociation into N adatoms at a high θ_N_ coverage is clearly exothermic, ranging from −0.60 (Ta_2_N) to −3.36 eV (Ta_2_C). Notice that, even though this last dissociative step may be endothermic, the analysis of the M_2_XN_2_ formation energies is constantly exothermic, see Figure 1, and *E*_form_ values displayed in Table S3 of the SI, going from −1.46 (Cr_2_CN_2_) to −4.28 eV (Ta_2_CN_2_). Thus, such M_2_XN_2_ MXenes are thermodynamically favorable, although, in light of the results, some reaction steps may be thermodynamically uphill, and in some cases, kinetically hindered, especially when reaching nearly full θ_N_ coverage situations. Notice, however, that even whenever N_2_ adsorption and/or dissociation is not favorable at such coverage limit, one may envision N-terminated domains, which may allow for a posteriori deposition of metal atoms.

**Figure 5:**
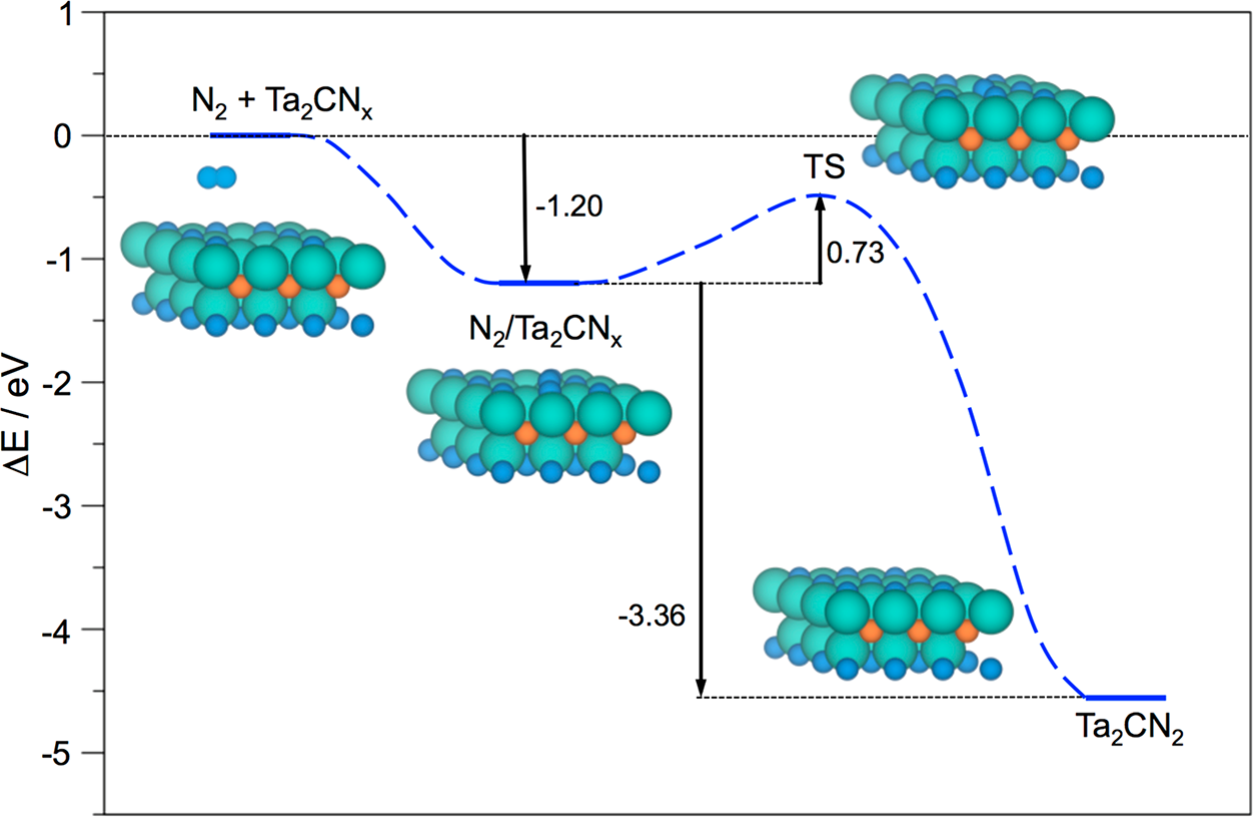
Reaction energy profile for N_2_ adsorption on the nearly full N-covered Ta_2_CN_*x*_ (0001) surface model and its subsequent dissociation into two N adatoms, overcoming the reaction step TS, to finally obtain the Ta_2_CN_2_ structure. The atomic models represent the different stages of the process. Color coding is the same as that in Figure 2.

**Figure 6:**
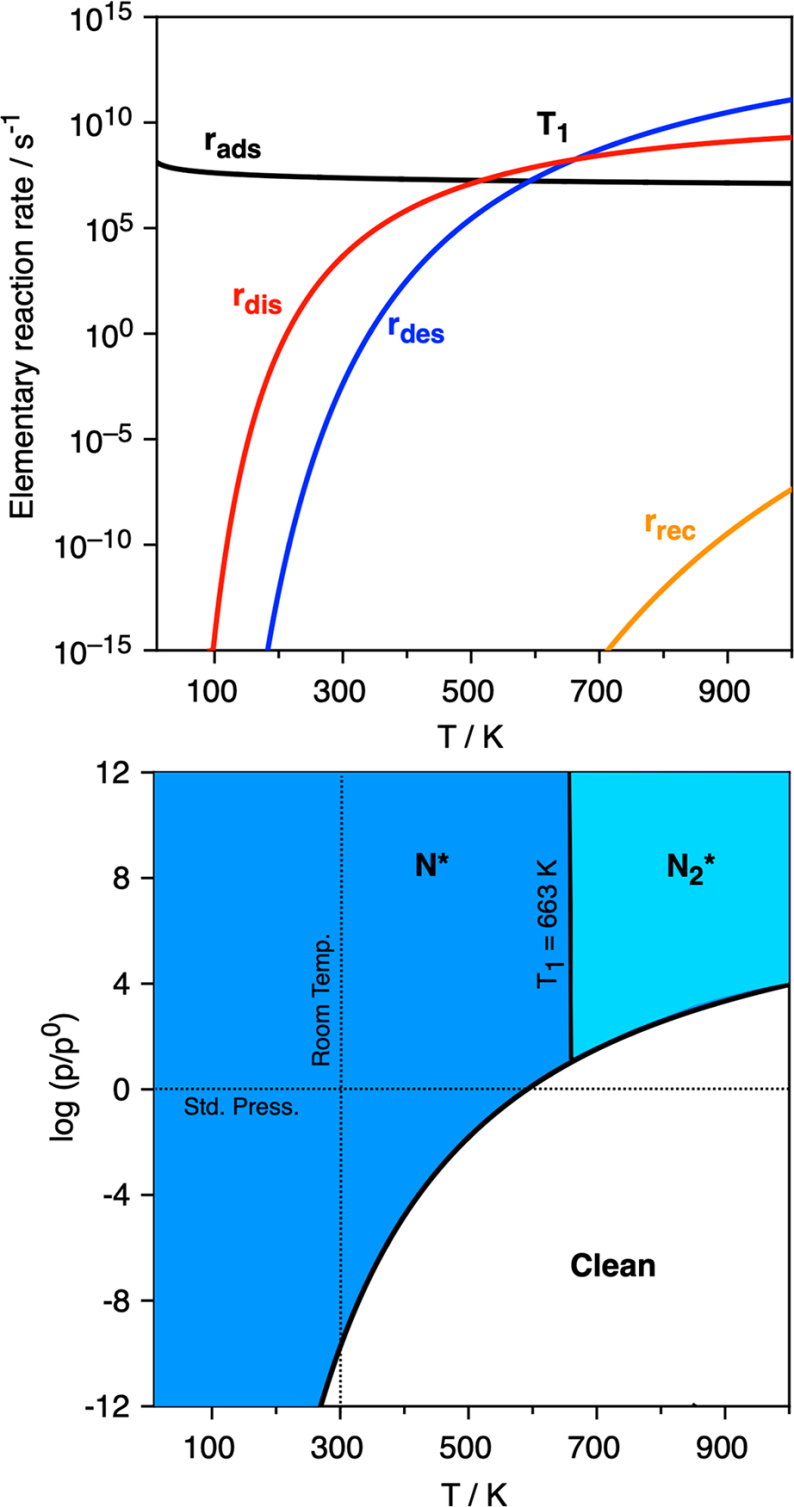
Top panel: Elementary reaction rates on the nearly fully N-covered Ta_2_C (0001) surface for the N_2_ adsorption at 0.1 bar as a function of temperature, *T*. The quantities *r*_ads_, *r*_des_, *r*_dis_, and *r*_rec_ are defined in Figure 3. Notice that at the *T*_1_ temperature of 663 K, *r*_dis_ = *r*_des_, and from this temperature upward, the desorption rate is larger than the N_2_ dissociation rate. Bottom panel: Kinetic phase diagram for N_2_ adsorption and dissociation as a function of N_2_ partial pressure, *p*_N_2__, and *T*. Dark and light blue colored areas reveal regions of preference toward N* and N_2_* moieties, respectively.

Taking again the Ta_2_C seed example shown in Figure 2, the reaction profile for the N_2_ adsorption and dissociation on the nearly fully N-covered model is shown in Figure 5, here revealing an *E*_ads_^N_2_^ of −1.20 eV, still larger than the N_2_ dissociation energy barrier, *E*_b_^N_2_^, of 0.73 eV, leading to the formation of the Ta_2_CN_2_ (0001) surface, with a reaction energy, Δ*E*_dis_^N_2_^, of −3.36 eV, revealing how favorable the formation of Ta_2_CN_2_ is, with an *E*_form_ of −4.28 eV per structural unit. The elementary reaction rates are shown in Figure 6, alongside with the built kinetic phase diagram, revealing a somewhat smaller region for N_2_ adsorption compared to the pristine case shown in Figure 3. Furthermore, for low temperatures, *r*_dis_ is larger than *r*_des_, but there is a limit temperature, *T*_1_, of 663 K, from which the adsorbed N_2_* would more likely desorb than dissociate and, therefore, a preference toward having N_2_* is forecasted. Still, the N_2_ dissociation capability is featured, revealing that, at *p*_N_2__ = 1 bar, one could complete the N* full coverage at working temperatures below ∼594 K.

Having analyzed the possibility of acquiring N-terminated M_2_XN_2_ MXenes, the next step is to investigate whether they can finally become metal-covered. To this end, we first inspected the adsorption of a single M′ atom on M_2_XN_2_ MXenes, where M′ is one of the MXenes M constituents, that is, from group IV (Ti, Zr, Hf), V (V, Nb, Ta), or VI (Cr, Mo, W). The M′ adatom was adsorbed respecting the regular M_2_XN_2_ stacking; H_X_ sites for regular ABC stacking and H_M_ for regular ABA stacking. In the case of Janus endings, different hollow sites were evaluated, although there is a preference trend for M′ being adsorbed on hollows with no underlying atoms in the two first subsurface layers, which are H and H_C_ sites for H_C_ and H_M_ sides of Janus Ti_2_CN_2_, Zr_2_CN_2_, and Hf_2_CN_2_, and H_C_ and H sites for H and H_C_ N-endings of Cr_2_CN_2_. A complete list of sites and the corresponding *E*_ads_^M′^ values is compiled in Table S4 of the SI.

**Figure 7:**
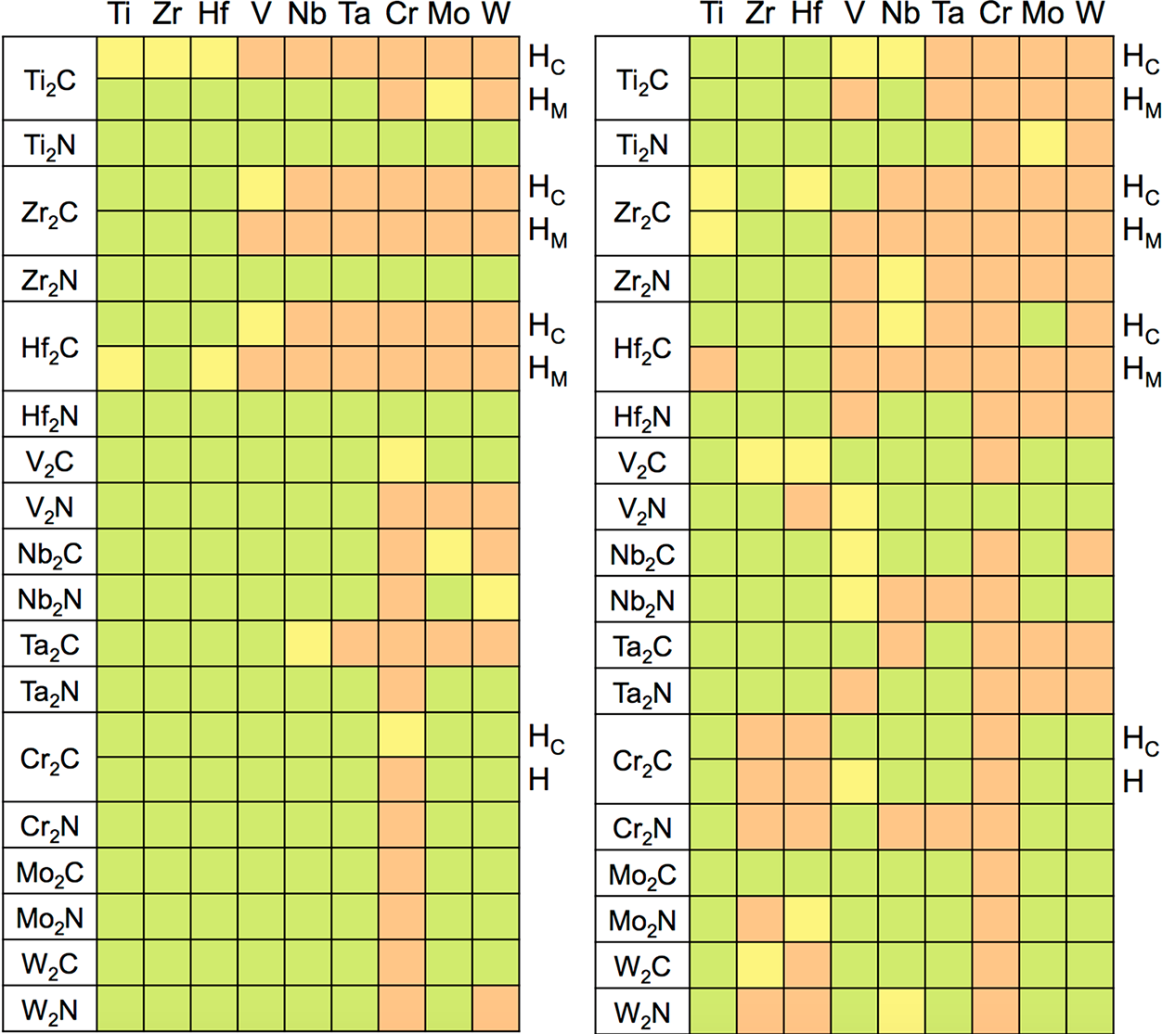
Degree of accomplishment of criteria *viii* and *ix*, implying M′ adsorption energies on pristine (left panel) or on nearly M′ fully covered (right panel) M_2_XN_2_ MXene models. Light green (red) color implies meeting (or not) the sought criterion, whereas light yellow color implies dubious cases within the DFT ± 0.2 eV accuracy range. H_C_ and H_M_ denote hollow sites where the adatoms are vertically aligned with a carbon or metal, respectively.

The definition of *E*_ads_^M′^ already implies that for negative values there is a preference for M′ to adsorb on the M_2_XN_2_ model rather than staying fully coordinated in the M′ bulk environment, and therefore, underscoring a thermodynamic preference for having such metals as isolated adatoms. This hints toward the possible use of M_2_XN_2_ as substrates for such M′ surface single atoms (SAs), employable as specially engineered atomic centers,^40^ as seen in the past for pristine MXenes and other 2D carbon-based materials,^32−34^ although the analysis of SAs on these MXenes is not the goal of the present paper. Therefore, we just mention that, in the explored cases, it seems clear that when going along the *d* series, the possibility of having isolated M′ adatoms becomes less favorable (see Figure 7), in which group VI M′ (Cr, Mo, W) starts to be unfavorable toward M′ adatom isolation. This is mostly due to the higher cohesive energies displayed by transition metals along the d series up to the *d*^5^ configurations, as a result of filling the bonding states of the *d*-band.^33,41^

However, the above is no rule of thumb, and the possibility of finding M′ isolated also depends on the considered M_2_XN_2_ substrate, with several cases being found where M′ single adatom adsorption is favorable. In particular, this happens for Ti_2_NN_2_, Zr_2_NN_2_, and Hf_2_NN_2_ group IV M_2_N MXenes and is closely followed by V_2_CN_2_. Also, the adsorption strength ranges from weak *E*_ads_^M′^ is −0.21 eV for Zr on the H_M_ side of Hf_2_CN_2_ to rather strong, with a value of −8.84 eV for Hf adatoms on W_2_CN_2_. Indeed, such highly exothermic values contrast with the adatom preference on pristine M_2_C (0001) surfaces of −0.95 eV at most for Sc adatoms on Cr_2_C, using the same models and computational setup as the ones employed here.^34^ As found on previous systematic studies on the transition metal adatoms’ stability on extended graphene and graphyne substrates,^33,41^ the M′ adsorption strength enhancement may well be related to the inherent substrate instability. This would explain why the M′ adsorption is weak on pristine M_2_X MXenes that, even if metastable, are experimentally reachable, whereas M′ adatoms can attach more strongly on N-terminated M_2_X models that, even if thermodynamically and kinetically driven, can be less stable, as experimentally only MXenes with −NH T_*x*_ have been isolated and characterized.^22^ Thus, such M_2_XN_2_ models have to be considered as intermediate cases, as would likely adsorb hydrogen, metal atoms, or other species so as to coat them, bestowing stability in this process.

In the search for M′_2_M_2_XN_2_, we next analyze the *E*_ads_^M′^ criterion for an almost full M′ surface coverage of ^8^/_9_ ML, thus featuring a single M′ vacancy on which the M′ is adsorbed in order to scrutinize whether the formation of a monolayer is preferred over M′ clustering, *i.e.*, favoring an epitaxial growth instead of the island/nanoparticle formation. The calculated values are compiled in Table S5 of the SI, and the results are visually summarized in Figure 7. As expected, both the exothermicity and the number of favorable cases decrease, given the lateral repulsions and the metal adlayer inherent tension to be commensurate to the M_2_XN_2_ seed. In general, and as seen in Figure 7, M′ adlayers are disfavored not only for group VI (Cr, Mo, W) but also in several group V (V, Nb, Ta) cases and even for certain group IV (Ti, Zr, Hf) situations. In any case, it is worth mentioning that, for all the M_2_X seeds and M_2_XN_2_ models, there is at least one M′ metal from which an adlayer formation would be thermodynamically driven, and that is why the low and high coverage *E*_ads_^M′^ criteria are generally met in Figure 1. The only exception to this is the H_C_ side of Ti_2_C, where the single adatom adsorption is dubious for Ti, Zr, and Hf (see Figure 7).

**Figure 8:**
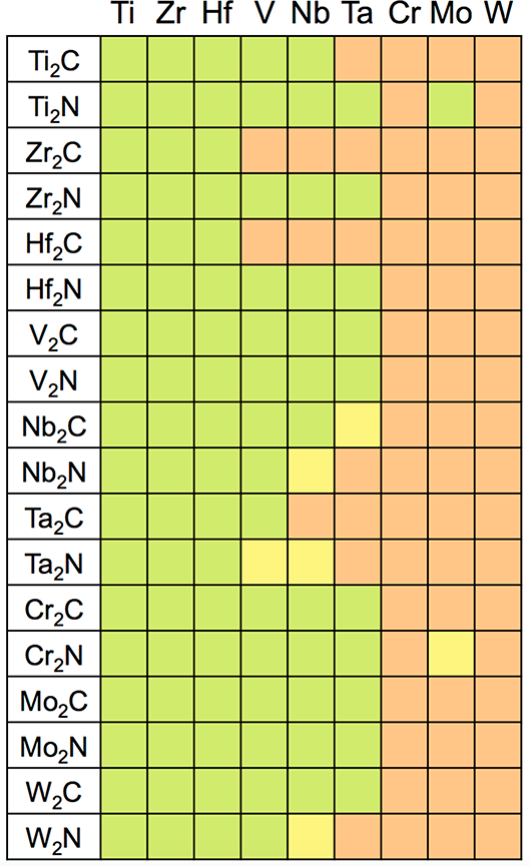
Degree of accomplishment of criteria *x*, *i.e.*, M′_2_M_2_XN_2_ MXenes formation energies. Light green (red) color implies meeting (or not) the sought criterion, while light yellow color implies dubious cases within the DFT ± 0.2 eV accuracy.

Finally, even if the first and last adatom adsorptions are thermodynamically possible during the formation of an M′ adlayer, the formation energies of the resulting surface, *E*_form_, are analyzed in the last decalogue criterion. The computed values are displayed in Table S6 of the SI, and graphically shown in Figure 8. From this figure, a clear pattern arises; the formation energies become more positive, and concomitantly thermodynamically less favored, when going along the M′ *d* series, *i.e.*, when moving from groups IV to VI, to the point of being generally not favorable for any group VI M′, except for the case of Mo_2_Ti_2_NN_2_, with an *E*_form_ of −0.35 eV. On the contrary, group IV M′ adlayers seem to be thermodynamically suited regardless of the M_2_X seed, with values ranging from mild, −0.49 eV for Zr on W_2_CN_2_, to highly exothermic, −4.43 eV for Ti on Ti_2_NN_2_, *i.e.*, the epitaxial synthesis of Ti_4_N_3_, encouragingly, coinciding with the first example of a stable two-dimensional transition metal nitride, which was attained experimentally through the selective etching of Al from a MAX phase precursor.^42^ This is again partly due to the above-mentioned larger cohesive energy encountered for transition metals when increasing the number of *d* electrons, at least up to *d*^5^ configurations, filling all the bonding states. Notice as well that, according to the present computational framework, the epitaxial synthesis of M_4_N_3_ MXenes should be feasible. This is an important issue since, despite having been forecasted to be more stable than M_4_C_3_, they have not yet been successfully synthesized, at variance with their C-based MXene counterparts.^2,43^ Notice, aside, that once an M′_2_M_2_XN_2_ MXene is obtained, it can be used as a further seed to obtain MXenes with larger *n*, and that, furthermore, given the similar chemical activity of M_*n*+1_X_*n*_ MXenes, seeds with *n* = 2, 3 could well be used for the epitaxial growth, as a way of obtaining *n* > 3 MXenes.

**Figure 9:**
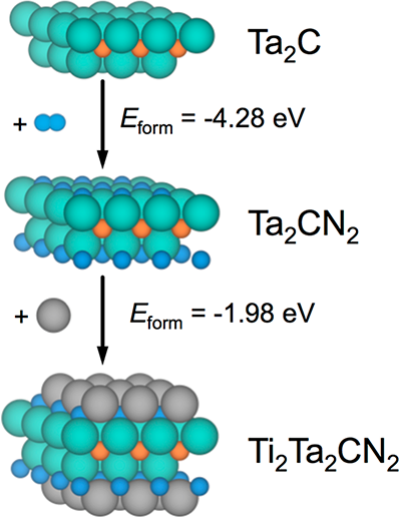
Steps of formation of Ta_2_CN_2_ and Ti_2_Ta_2_CN_2_ models departing from Ta_2_C seed, alongside with the formation energies for each step. Ti atoms are shown as gray spheres, whereas the rest of the color coding is the same as that in Figure 2.

Retaking the Ta_2_C (0001) seed prototypical case, Figures 2 and 5 showed the viability of having a fully N-covered situation, accomplishing both thermodynamic and kinetic thresholds. Furthermore, the formation energy of Ta_2_CN_2_ is −4.28 eV, thus strongly suggesting the stability of this N-terminated MXene. The adsorption of the first Ti adatom on Ta_2_CN_2_ features an *E*_ads_^M′^ of −1.32 eV with respect to Ti bulk, and a similar value of −1.29 eV is found for the completion of the Ti_2_Ta_2_CN_2_ MXene, whose formation energy with respect to Ta_2_CN_2_ and bulk Ti is −1.98 eV, see Figure 9. This is just one example out of the many studied showing how the decalogue of criteria for MXene epitaxial growth is accomplished, meeting all the required stability and kinetic thresholds. Actually, one could foresee, *e.g.*, the use of the as-synthesized Ti_2_Ta_2_CN_2_ MXene as a seed to grow thicker MXenes, even mixing different metallic compositions at different materials strata. Indeed, as a proof of concept, the N_2_ adsorption energy on Ti_2_Ta_2_CN_2_ was computed to be −3.19 eV, nicely lying in between the values for Ta_2_C (seed) and Ti_2_N (growth), of −2.35 and −3.45 eV, respectively, succinctly unfolding that the tailored epitaxial growth on selected MXene seeds can be used as a way of fine-tuning the MXene surface chemical activity, and enlarges the *o*-MXenes family offering a way to control its layer composition.

**Figure 10:**
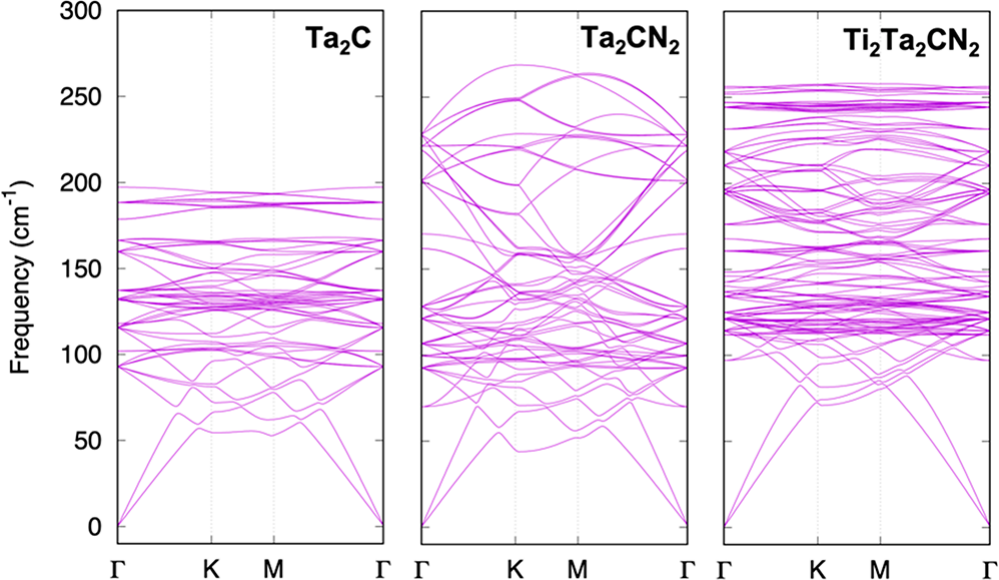
Phonon dispersion curves for Ta_2_C, Ta_2_CN_2_, and Ti_2_Ta_2_CN_2_ MXenes, along the main **Γ** → **M** → **K** → **Γk**-path in the Brillouin zone of the reciprocal space.

One should bear in mind, though, that even when all the decalogue criteria are met, other aspects may determine whether a certain MXene or *o*-MXene could be epitaxially grown as foreshown. For instance, the MXene M_2_X seed, the intermediate M_2_XN_2_, and the final resulting M′_2_M_2_XN_2_ MXene should be dynamically stable. Actually, many pristine and functionalized MXenes have been shown to be dynamically stable in the literature.^44−48^ In the present case, we explored the dynamic stability on the exemplary case of Ta_2_C seed, the Ta_2_CN_2_ intermediate, and the final Ti_2_Ta_2_CN_2_ MXene by appropriate phonon calculations. The results are summarized in Figure 10, where acoustic and optical vibrational modes dispersions in the **k**-space —along main **Γ** → **M** → **K** → **Γk**-paths with a convergence criterion of 10^–8^ eV— have been gained through density functional perturbation theory (DFPT) as implemented in the PHONOPY code.^49^ The dynamical stability of such cases is clear from the absence of imaginary frequencies in the selected **k**-path. Even if proven in such an exemplary case, such assessment would be useful for other systems, although the previous studies and the present evaluation seem to guarantee that there will be a number of situations where the epitaxial growth would be feasible.

Another aspect to regard beyond the dynamic stability is whether the explored M′_2_M_2_XN_2_ MXenes would actually remain layered as designed, or whether in-plane alloying of M′ and M could be expected, as observed experimentally in the so-called *i*-MXenes.^44,50^ To at least address this point on the exemplary case of Ti_2_Ta_2_CN_2_, different arrangements were evaluated assuming the same overall stoichiometry, but having equal parts of Ti and Ta on the metal layers. The results, summarized in Figure S3 of the SI, reveal that any *i*-MXene alloy configuration is higher in energy, with differences in energy, Δ*E*, ranging from 0.01 to 0.15 eV, referred to a *p*(2×2) unit cell of Ti_2_Ta_2_CN_2_. Given that some cases are basically isoenergetic, the interchange of surface Ti with subsurface Ta atom was further investigated, but the sequential interchange of Ti↔N↔Ta leads to either situations very high in energy, above 8 eV, or to jeopardized MXene structures. Thus, the only viable way of having metal disorder is through the presence of vacancies. Indeed, the surface Ti diffusing to a subsurface Ta vacancy is thermodynamically favorable by −1.98 eV, but still surmounting a costly energy barrier of 2.26 eV. Only when having vicinal Ta and N vacancies, the Ti diffusion is energetically favorable by −2.28 eV, with a moderate energy barrier of 0.60 eV. However, it must be borne in mind that having such vicinal vacancies are energetically costly, with a formation energy calculated to be of 5.28 eV with respect Ta bulk and N_2_ gas. Even if spontaneous alloying in Ti_2_Ta_2_CN_2_ seems unlikely, the situation may change for other mixtures, a point to be regarded in future studies. However, the metal alloying on epitaxially grown MXenes is something worthy to be analyzed, and that even can be prompted, by depositing given quantities of a couple of metals, a point that widens the possibilities of manufacturing different MXenes by epitaxial growth.

## Conclusions

In summary, we provided here an atomistic radiography of the plausible epitaxial growth of MXenes by depositing alternating layers of N and early transition metal atoms on a set of 18 carbide and nitride M_2_X MXene seeds, considering the energetically most stable stacking conformation, and scrutinizing a decalogue of thermodynamic and kinetic energy criteria of different growth steps by first-principles calculations.

The results show that, while pristine M_2_X (0001) surfaces systematically easily adsorb and dissociate N_2_, the formation of fully N-covered M_2_XN_2_ models may be kinetically hindered depending on the employed M_2_X seed, being thus the most restrictive criterion and reaction step, even though the formation energy of M_2_XN_2_ models is thermodynamically advantageous. In the case of group IV Ti_2_C, Zr_2_C, and Hf_2_C, as well as of Cr_2_C, different surface N-endings are found, rendering these M_2_XN_2_ MXenes effectively Janus structures.

The formation of early transition metal additional adlayers leading to M′_2_M_2_XN_2_ MXenes is, in general, thermodynamically driven for all the M_2_XN_2_ models, although there are exceptions to this trend. In any case, a route map in the epitaxial growth of MXenes by alternating N and metal adlayers is provided, based on which further casts can be made. Furthermore, the presented epitaxial growth is introduced as a way of achieving the M_4_N_3_ synthesis, obtaining wider MXenes going beyond seven layers, and/or tuning the MXene formulation, generating *o*-MXenes at will. The present work is expected to trigger future experimental work aiming at obtaining alternative MXenes by exploiting the epitaxial growth as here analyzed and proposed.

## Methods

A set of MXene seeds with M_2_X formula has been studied, including M elements from groups IV (Ti, Zr, Hf), V (V, Nb, Ta), and VI (Cr, Mo, W), whereas X = C or N, totaling 18 M_2_X MXenes. Their basal (0001) surfaces have been described through *p*(3×3) supercell slab systems, as done in former studies.^6,51,52^ In all calculations, the MXene atomic positions as well as those of adsorbate atoms or molecules were fully optimized by total energy minimization. The employed supercells have a vacuum length along the (0001) basal cell vector of at least 10 Å, enough to practically nullify the interactions among replicated slabs or the adsorbates. Notice that M_2_X, M_2_XN_2_, and M′_2_M_2_XN_2_ MXenes unit cells were fully optimized prior to adsorbing either metal atoms thereon or adsorbing and dissociating a N_2_ molecule.

One aspect to be regarded is that M_2_X MXene seeds do not necessarily have to feature the ABC atomic layer stacking of the parent MAX phases, as recent studies revealed that ABA stacking is thermodynamically preferred and kinetically achievable for some MXenes. Indeed, the ABA preference is fostered by the number of the *d* electrons of the M element, when having X = N instead of C, and when T_*x*_ = O.^53^ Furthermore, in this previous computational study, it was emphasized that stacking could have an impact on the surface chemical activity, evaluated for the N_2_ adsorption and dissociation, with changes up to ∼1 eV for *E*_ads_ and up to 0.3 eV for *E*_b_. Accordingly, in this work, the most stable stacking was used in each M_2_X seed, and a stacking energetic preference study was conducted for the N-terminated cases, *i.e.*, M_2_XN_2_.

The present first-principles-based analysis was carried out using the Vienna *ab initio* simulation package (VASP).^54^ In order to compare the present results with previous studies on N_2_ adsorption and dissociation on M_2_X (0001) surfaces,^28,53^ the Perdew–Burke–Ernzerhof (PBE) exchange-correlation functional,^55^ combined with the Grimme D3 treatment of dispersive forces,^56^ was employed. Projector-augmented wave (PAW) pseudopotentials were used to describe the effect of core electrons on the valence electron density.^57^ The latter was expanded in a plane wave basis set with a maximum kinetic energy cutoff of 415 eV. The Brillouin zone integration was sampled using optimal Monkhorst–Pack **k**-grids of 5×5×1 dimensions.^58^ The threshold for the electronic optimization has been set to 10^–5^ eV, and geometry optimizations were considered finished when forces acting on atoms were below 0.01 eV Å^–1^. Notice that test calculations using more stringent values for kinetic energy cutoff, **k**-points mesh, or the thresholds used as optimization criteria yielded variations in computed *E*_ads_ below chemical accuracy, *i.e.*, below ∼0.04 eV —1 kcal·mol^–1^. All calculations were carried out accounting for spin polarization unless stated otherwise. Still, test calculations on *p*(1×1) unit cells considering nonmagnetic, ferromagnetic, and antiferromagnetic solutions confirmed that the energy difference between them is almost negligible for M_2_X seeds, with no magnetization found neither for M_2_XN_2_ nor for M′_2_M_2_XN_2_ materials (see Table S7 of the SI). To evaluate the stability of the epitaxially grown materials, N_2_ in vacuum and bulk metal references were taken as references. The geometry of the isolated N_2_ molecule in its closed shell electronic ground state was optimized using the PBE-D3 functional at Γ-point in an asymmetric cell of 9×10×11 Å, as previously done in the literature.^28^ Bulk metals were optimized as done and available in the literature.^59−61^

The search for the adsorbed N_2_ dissociation transitions states (TSs) was carried out by means of either the climbing-image nudged elastic band (CI-NEB) method,^62^ or the dimer^63^ methods. All stationary points, that is, the found minima and TSs, were properly characterized through a pertinent vibrational frequency analysis, where frequencies were calculated only for the adsorbate, and hence, decoupled from the substrate phonons. To estimate the frequencies, the corresponding block of the Hessian matrix was built through finite differences of analytical gradients with displacements of 0.015 Å. The resulting matrix was diagonalized, and the so-gained eigenvalues correspond to the frequencies of the normal vibrational modes. All minima and TSs featured none or just one imaginary frequency, respectively.

## Supporting Information Available

N_2_ adsorption energies, dissociation energy barriers, and dissociation energy changes on pristine and nearly fully N-covered M_2_X (0001) MXene models; M_2_XN_2_ formation energies and differences in energy between ABC and ABA stacking conformations for M_2_X and M_2_XN_2_ models; M′ adsorption energies on M_2_XN_2_ models and nearly M′ fully covered M_2_XN_2_ models; M′_2_M_2_XN_2_ formation energies; difference in energies for diverse alloys of Ti_2_Ta_2_CN_2_ MXene model; difference in energies between nonmagnetic, ferromagnetic, and antiferromagnetic solutions (PDF)
