# Supplementary material for: MXenes à la Carte: Tailoring the Epitaxial Growth Alternating Nitrogen and Transition Metal Layers
Source: ACS Nano. 2022 Jul 22;16(8):12541–52. doi: 10.1021/acsnano.2c04029 (PMC9881142; doi:10.1021/acsnano.2c04029)
Supplement: Supplementary file 1 — nn2c04029_si_001.pdf [file nn2c04029_si_001.pdf]

MXenes *à la Carte*: Tailoring the Epitaxial Growth Alternating Nitrogen and Transition Metal Layers

José D. Gouveia,[‡] Ángel Morales-García,[†] Francesc Viñes[*,†] José R. B. Gomes,[‡] Francesc Illas[†]

[†]*Departament de Ciència de Materials i Química Física & Institut de Química Teòrica i Computacional (IQTCUB), Universitat de Barcelona, c/ Martí i Franquès 1-11, 08028 Barcelona, Spain*

[‡]*CICECO – Aveiro Institute of Materials, Department of Chemistry, University of Aveiro, Campus Universitário de Santiago, 3810-193 Aveiro, Portugal*

[*] Corresponding author: Francesc Viñes (francesc.vines@ub.edu)

Table S1. N_2 adsorption energies, $E_{\mathrm{ads}}^{\mathrm{N_2}}$, adsorbed N_2 dissociation energy barriers, $E_{\mathrm{b}}^{\mathrm{N_2}}$, and N_2 dissociation reaction step energies, $\Delta E_{\mathrm{dis}}^{\mathrm{N_2}}$, as estimated on pristine M_2X (0001) models.

**M_2X (0001)**	**Stacking**	$E_{\mathrm{ads}}^{\mathrm{N_2}}$ **/ eV**	$E_{\mathrm{b}}^{\mathrm{N_2}}$ **/ eV**	$\Delta E_{\mathrm{dis}}^{\mathrm{N_2}}$ **/ eV**
**Ti_2C**	ABC	-3.26	0.98	-1.55
**Ti_2N**	ABC	-3.45	0.93	-2.00
**Zr_2C**	ABC	-2.84	1.10	-1.60
**Zr_2N**	ABC	-3.08	0.91	-2.25
**Hf_2C**	ABC	-3.14	0.96	-2.02
**Hf_2N**	ABC	-3.30	0.75	-2.61
**V_2C**	ABC	-2.99	0.80	-1.77
**V_2N**	ABC	-2.10	0.78	-2.20
**Nb_2C**	ABC	-2.41	0.75	-2.18
**Nb_2N**	ABA	-2.68	0.78	-2.00
**Ta_2C**	ABC	-2.35	0.53	-2.72
**Ta_2N**	ABA	-2.85	0.54	-2.38
**Cr_2C**	ABA	-2.06	0.78	-1.37
**Cr_2N**	ABA	-1.99	0.61	-1.89
**Mo_2C**	ABA	-1.36	0.62	-2.16
**Mo_2N**	ABA	-1.73	0.41	-2.60
**W_2C**	ABA	-1.14	0.37	-2.53
**W_2N**	ABA	-1.27	0.18	-3.22

Figure S1. Linear regression of $E_{ads}^{N_2}$ on bare M_2X (0001) surfaces *vs.* (top) *d*-band center, ε_d, and (bottom) surface metal charges, ΔQ_m. The regression coefficients, R, are given for each plot, and the linear equations are $E_{ads}^{N_2}$= -0.324·ε_d – 1.945, and $E_{ads}^{N_2}$= -3.659·ΔQ_m + 0.967.

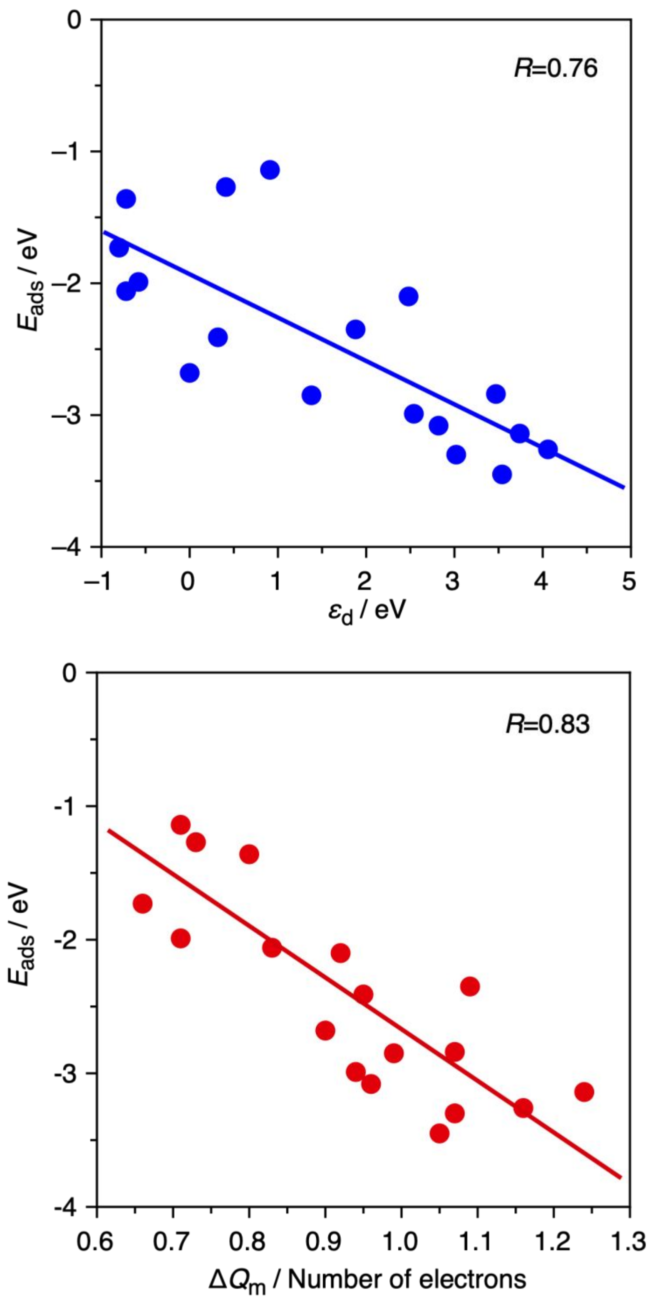

Figure S2. Brønsted–Evans–Polanyi (BEP) linear relation between N_2 dissociation energy barriers, E_b, and the reaction step change in energy, ΔE_{dis}. The regression coefficients, *R*, is estimated to be 0.96, while the linear equations is $E_b = 0.70 \cdot \varepsilon_d + 2.33$, with a mean absolute error of 0.3 eV.

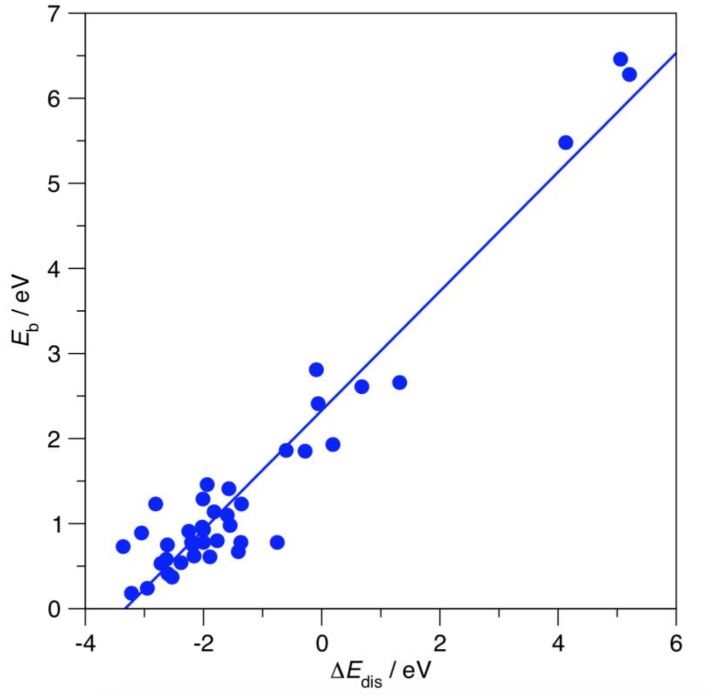

Table S2. Difference in energy between ABC and ABA stacking of the core M_2X in M_2XN_2, ΔE_{stack}, given per formula unit. A negative ΔE_{stack} value implies a preference towards ABA stacking in the core M_2X seed. The full stacking sequence is also displayed, and notice Janus endings in Ti_2CN_2, Zr_2CN_2, Hf_2CN_2, and Cr_2CN_2.

M_2XN_2	Stacking	ΔE_{stack} / eV
Ti_2CN_2	BABCA	0.65
Ti_2NN_2	CABCA	0.87
Zr_2CN_2	BABCA	0.83
Zr_2NN_2	CABCA	1.06
Hf_2CN_2	BABCA	0.75
Hf_2NN_2	CABCA	1.00
V_2CN_2	CABCA	0.68
V_2NN_2	CABCA	0.50
Nb_2CN_2	CABCA	1.24
Nb_2NN_2	CABCA	0.21
Ta_2CN_2	CABCA	1.59
Ta_2NN_2	CABCA	0.50
Cr_2CN_2	BABAC	-0.21
Cr_2NN_2	BABAB	-0.36
Mo_2CN_2	BABAB	-0.30
Mo_2NN_2	BABAB	-0.56
W_2CN_2	BABAB	-0.33
W_2NN_2	BABAB	-0.31

Table S3. N_2 adsorption energies, $E_{\mathrm{ads}}^{\mathrm{N_2}}$, adsorbed N_2 dissociation energy barriers, $E_{\mathrm{b}}^{\mathrm{N_2}}$, and N_2 dissociation reaction step energies, $\Delta E_{\mathrm{dis}}^{\mathrm{N_2}}$, as estimated on nearly fully N-covered M_2X (0001) models, nominally at a N adatom coverage, θ_N, of $^7/_9$ of a monolayer (ML). Aside, the computed formation energies, E_{form}, of the M_2XN_2 models.

M_2XN_2 (0001)	Stacking	Site	$E_{\mathrm{ads}}^{\mathrm{N_2}}$ / eV	$E_{\mathrm{b}}^{\mathrm{N_2}}$ / eV	$\Delta E_{\mathrm{dis}}^{\mathrm{N_2}}$ / eV	E_{form} / eV
**Ti_2C**	BABCA	H_C	-0.90	1.41	-1.57	-3.44
		H_M	-1.02	1.46	-1.94	
**Ti_2N**	CABCA	H_M	-0.45	6.28	5.21	-2.90
**Zr_2C**	BABCA	H_C	-1.46	1.23	-1.36	-3.41
		H_M	-1.58	1.23	-2.81	
**Zr_2N**	CABCA	H_M	-0.43	6.46	5.06	-3.39
**Hf_2C**	BABCA	H_C	-1.64	1.14	-1.82	-3.97
		H_M	-1.05	0.89	-3.05	
**Hf_2N**	CABCA	H_M	-0.53	5.48	4.13	-4.02
**V_2C**	CABCA	H_M	-0.20	1.85	-0.28	-2.45
**V_2N**	CABCA	H_M	-0.87	0.67	-1.41	-2.76
**Nb_2C**	CABCA	H_M	-0.90	1.29	-2.01	-3.27
**Nb_2N**	CABCA	H_M	-1.30	0.24	-2.95	-3.82
**Ta_2C**	CABCA	H_M	-1.20	0.73	-3.36	-4.28
**Ta_2N**	CABCA	H_M	-2.05	1.86	-0.60	-3.97
**Cr_2C**	BABAC	H_C	-1.65	2.81	-0.09	-1.46
		H	0.50	0.58	-2.63	
**Cr_2N**	BABAB	H_C	-0.17	2.66	1.32	-1.84
**Mo_2C**	BABAB	H_C	0.97	0.78	-0.75	-1.63
**Mo_2N**	BABAB	H_C	-0.10	1.93	0.19	-2.35
**W_2C**	BABAB	H_C	-0.17	2.61	0.68	-1.86
**W_2N**	BABAB	H_C	-0.18	2.41	-0.06	-2.74

Table S4. M' adsorption energies, $E_{\text{ads}}^{\text{M}'}$, in eV, on pristine M_2XN_2 models defined in Table S3, as well as the preferred M' adsorption site.

M_2XN_2	Surface	M' Site	Ti	Zr	Hf	V	Nb	Ta	Cr	Mo	W
**Ti_2C**	H_C	H	-0.17	-0.09	-0.12	0.44	0.55	0.75	3.52	1.40	1.93
	H_M	H_C	-2.41	-2.47	-2.39	-1.11	-1.07	-0.78	1.89	-0.12	0.58
**Ti_2N**	H_M	H_N	-8.08	-8.19	-8.23	-7.48	-7.66	-7.59	-4.32	-6.80	-6.41
**Zr_2C**	H_C	H	-0.62	-0.53	-0.62	0.09	0.27	0.33	3.37	1.29	1.58
	H_M	H_C	-0.72	-0.79	-0.69	0.54	0.66	0.95	3.48	1.42	2.08
**Zr_2N**	H_M	H_N	-8.00	-8.14	-8.18	-7.66	-7.83	-7.80	-4.46	-6.98	-6.70
**Hf_2C**	H_C	H	-0.63	-0.55	-0.65	0.08	0.29	0.35	3.41	1.34	1.62
	H_M	H_C	-0.15	-0.21	-0.18	1.10	1.21	1.30	3.70	1.66	2.44
**Hf_2N**	H_M	H_N	-8.38	-8.58	-8.66	-7.47	-7.92	-7.97	-4.26	-6.79	-6.55
**V_2C**	H_M	H_C	-3.61	-3.56	-3.57	-2.37	-2.28	-1.99	0.71	-1.27	-0.55
**V_2N**	H_M	H_N	-3.80	-3.67	-3.60	-2.92	-2.92	-2.43	0.24	-1.66	-0.89
**Nb_2C**	H_M	H_C	-2.30	-2.36	-2.23	-1.17	-0.78	-0.61	2.01	-0.05	0.53
**Nb_2N**	H_M	H_N	-3.16	-3.76	-2.81	-2.16	-2.17	-1.49	1.07	-0.81	-0.05
**Ta_2C**	H_M	H_C	-1.32	-1.27	-1.21	-0.27	0.04	0.39	2.53	0.55	1.42
**Ta_2N**	H_M	H_N	-4.02	-4.19	-4.22	-2.46	-2.42	-2.33	1.05	-1.08	-0.82
**Cr_2C**	H_C	H	-5.12	-5.12	-5.07	-3.84	-3.56	-3.56	-0.08	-2.07	-1.69
	H	H_C	-5.24	-5.16	-4.98	-2.83	-4.80	-4.62	0.81	-1.17	-0.63
**Cr_2N**	H_C	H_M	-4.59	-4.65	-4.53	-2.98	-2.56	-2.57	0.81	-1.05	-0.49
**Mo_2C**	H_C	H_M	-4.60	-4.73	-4.61	-3.07	-2.80	-2.83	0.65	-1.38	-0.92
**Mo_2N**	H_C	H_M	-4.64	-4.81	-4.69	-2.82	-2.49	-2.51	1.05	-0.80	-0.23
**W_2C**	H_C	H_M	-4.62	-4.82	-8.84	-6.66	-3.13	-2.95	0.35	-1.69	-0.99
**W_2N**	H_C	H_M	-4.11	-4.32	-4.36	-2.18	-2.06	-1.95	1.46	-0.32	0.55

Table S5. M' adsorption energies, $E_{ads}^{M'}$, in eV, on nearly fully M' covered $M'_2M_2XN_2$, as well as the preferred M' adsorption site.

M_2XN_2	Surface	M' Site	Ti	Zr	Hf	V	Nb	Ta	Cr	Mo	W
**Ti_2C**	H_C	H	-1.45	-1.08	-1.28	-0.15	0.08	6.32	2.71	0.74	1.65
	H_M	H_C	-1.14	-1.20	-1.29	0.42	-0.66	4.65	2.78	0.45	3.79
**Ti_2N**	H_M	H_N	-1.73	-1.10	-1.47	-0.66	-1.37	-1.39	3.17	0.03	0.44
**Zr_2C**	H_C	H	-0.06	-1.18	-0.03	-2.24	0.48	1.74	6.16	1.80	3.20
	H_M	H_C	-0.01	-0.96	-0.59	1.51	0.62	0.83	8.73	0.61	4.31
**Zr_2N**	H_M	H_N	-0.40	-1.70	-1.51	1.74	-0.18	0.48	3.55	1.42	2.29
**Hf_2C**	H_C	H	-0.32	-1.02	-0.76	5.34	-0.06	1.26	5.60	-1.54	1.95
	H_M	H_C	0.25	-1.00	-0.77	4.38	0.59	2.63	4.53	0.57	2.67
**Hf_2N**	H_M	H_N	-1.15	-1.35	-1.62	0.40	-1.08	-0.85	3.77	0.86	1.74
**V_2C**	H_M	H_C	-1.35	-0.18	-0.04	-0.91	-1.53	-1.51	3.08	-1.02	-1.05
**V_2N**	H_M	H_N	-4.37	-0.37	0.31	-0.20	-1.23	-1.01	-0.29	-0.75	-0.45
**Nb_2C**	H_M	H_C	-1.34	-1.15	-1.40	-0.06	-0.42	-0.35	2.34	-2.26	1.27
**Nb_2N**	H_M	H_N	-1.48	-0.88	-1.18	0.19	0.53	0.43	3.56	-2.44	-1.78
**Ta_2C**	H_M	H_C	-1.29	-0.65	-0.67	-1.42	13.50	-0.63	3.67	1.12	9.38
**Ta_2N**	H_M	H_N	-0.26	-0.60	-0.46	5.63	-0.90	-0.71	9.37	1.09	2.29
**Cr_2C**	H_C	H	-0.41	0.16	0.51	-0.64	-0.97	-1.23	2.58	-0.74	-1.00
	H	H_C	-0.44	0.75	0.95	-0.02	-0.89	-0.92	3.82	-0.68	-0.97
**Cr_2N**	H_C	H_M	-0.94	1.69	1.48	-1.81	0.82	0.20	1.15	-0.71	-0.86
**Mo_2C**	H_C	H_M	-0.86	-0.86	-0.86	-0.63	-1.70	-2.06	4.75	-0.63	-1.05
**Mo_2N**	H_C	H_M	-1.02	0.36	0.18	-1.69	-0.51	-1.17	2.21	-0.86	-1.12
**W_2C**	H_C	H_M	-0.90	0.06	0.29	-0.94	-0.78	-1.23	2.84	-1.00	-1.04
**W_2N**	H_C	H_M	-0.63	1.21	0.82	-1.29	0.03	-0.42	2.31	-0.57	-0.61

Table S6. $M'_2M_2XN_2$ formation energies, E_{form}, in eV, as well as the stacking conformation.

M_2XN_2	Stacking	Ti	Zr	Hf	V	Nb	Ta	Cr	Mo	W
**Ti_2C**	CBABCAB	-2.85	-2.60	-2.81	-0.61	-0.40	0.07	5.28	0.88	2.06
**Ti_2N**	BCABCAB	-4.43	-3.93	-4.12	-2.48	-2.44	-2.09	3.81	-0.36	0.88
**Zr_2C**	CBABCAB	-1.39	-2.11	-1.96	0.74	0.31	0.66	7.18	2.01	3.20
**Zr_2N**	BCABCAB	-2.88	-3.52	-3.30	-0.43	-1.08	-0.29	5.35	0.90	2.42
**Hf_2C**	CBABCAB	-1.35	-1.96	-1.89	1.24	0.37	0.83	6.91	1.97	3.08
**Hf_2N**	BCABCAB	-3.32	-3.52	-3.55	-1.10	-1.42	-0.96	5.15	0.73	2.04
**V_2C**	BCABCAB	-3.61	-2.15	-2.37	-2.00	-1.37	-1.06	4.33	0.29	1.13
**V_2N**	BCABCAB	-4.27	-1.75	-1.95	-1.55	-0.87	-0.56	3.90	0.82	1.69
**Nb_2C**	BCABCAB	-2.94	-2.71	-2.76	-0.81	-0.30	-0.14	5.13	0.58	1.35
**Nb_2N**	BCABCAB	-2.71	-2.27	-2.36	-0.63	0.18	0.28	5.69	0.92	1.69
**Ta_2C**	BCABCAB	-1.98	-1.38	-1.59	-3.08	0.53	0.82	6.28	2.54	3.72
**Ta_2N**	BCABCAB	-2.17	-2.17	-2.21	0.15	-0.15	0.22	6.01	2.12	3.36
**Cr_2C**	CBABACB	-3.32	-1.73	-1.78	-1.83	-0.73	-0.77	4.19	0.60	1.07
**Cr_2N**	ABABABA	-3.15	-1.21	-1.10	-2.22	-0.53	-0.60	4.17	0.18	0.63
**Mo_2C**	ABABABA	-3.13	-3.13	-2.93	-1.29	-1.46	-1.55	5.60	0.45	0.97
**Mo_2N**	ABABABA	-2.72	-1.73	-1.61	-1.33	-0.43	-0.60	5.42	0.89	1.33
**W_2C**	ABABABA	-3.01	-2.32	-1.35	-0.57	-1.52	-1.36	4.89	0.03	0.76
**W_2N**	ABABABA	-1.70	-0.49	-0.61	-0.42	0.17	0.37	5.84	1.42	2.18

Figure S3. $Ti_2Ta_2CN_2$ optimized *o*-MXene model (bottom) and the difference in energy, Δ*E*, referred to a *p*(2×2) unit cell of $Ti_2Ta_2CN_2$, with different *i*-MXenes, having equal quantities of Ti and Ta on each metal layer. Ta, Ti, C, and N atoms represented by light green, grey, light brown, and blue spheres, respectively.

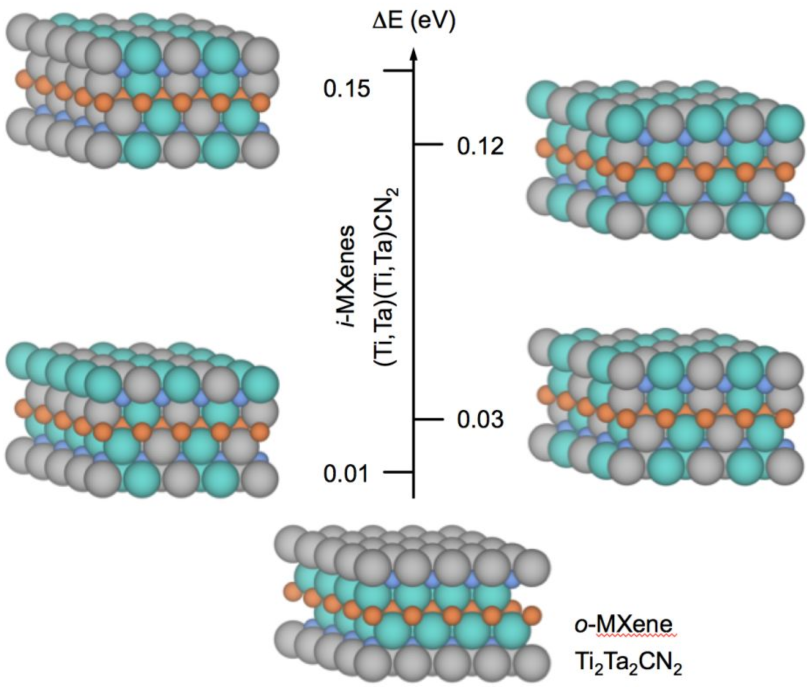

Table S7. Energy difference, Δ*E*, in eV, between ferromagnetic (FM) and antiferromagnetic (AFM) solutions relative to the non-magnetic (NM) one for the M_2C MXenes exhibiting a magnetic ground state. The predicted atomic magnetic moment on the two metal atoms of the *p*(1×1) MXene cell unit, μ_B^{at}, in Bohr magnetons, are given for both FM and AFM.

MXene	Δ*E*(FM)	μ_B^{at} (FM)	Δ*E*(AFM)	μ_B^{at} (AFM)
Ti_2C	-0.19	0.61 / 0.61	-0.34	0.68 / -0.68
Zr_2C	-0.23	0.47 / 0.47	-0.31	0.51 / -0.51
Hf_2C	-0.19	0.48 / 0.48	-0.25	0.50 / -0.50
